# Laccase-Mediated Fabrication of Food Packaging Films: A Critical Review of Functional Performance, Safety, and Industrial Viability

**DOI:** 10.3390/biom16091285

**Published:** 2026-09-05

**Authors:** Alessandro D’Annibale, Rosita Marabottini

**Affiliations:** Department for Innovation in Biological, Agro-Food and Forest Systems (DIBAF), University of Tuscia, Via S. Camillo De Lellis snc, 01100 Viterbo, Italy; marabottini@unitus.it

**Keywords:** laccase, food packaging, enzymatic crosslinking, biopolymers, active packaging, time-temperature indicators, nanozymes

## Abstract

Although natural biopolymers represent promising sustainable packaging alternatives, their weak mechanical and barrier properties limit industrial use. While previous reviews focus on descriptive aspects of enzymatic modification, this review fills a critical literature gap by systematically bridging molecular-level laccase-driven reactions with quantitative techno-economic and safety and regulatory frameworks. We evaluate the kinetic and topological differences between direct tyrosyl-coupled protein homopolymerisation and mediator-assisted ‘graft-then-link’ polysaccharide strategies. Crucially, we analyse how entrapment versus surface-immobilised architectures dictate mass-transfer regimes, establishing their specific functional fitness for active oxygen scavenging or intelligent time-temperature monitoring. Beyond physical performance, we critically assess the translational bottlenecks currently hindering industrial scaling. For the first time, we integrate a quantitative techno-economic analysis using the Technology Readiness Level (TRL) framework, demonstrating that active film fabrication costs (EUR 0.01–0.10/m^2^) are heavily offset by high-protein food waste savings (>EUR 2.00/kg). Finally, we navigate European and US regulatory landscapes for enzymatically active materials and evaluate safety risks via the Threshold of Toxicological Concern (TTC) model and deterministic migration modelling. This comprehensive analysis establishes a ‘Safe-by-Design’ paradigm, guiding the scalable development of intrinsically safe, high-performance biocatalytic packaging.

## 1. Introduction

The primary function of food packaging is to establish robust chemical, biological, and physical barriers that extend shelf life and mitigate global food waste [1]. Although synthetic petroleum-based plastics have historically met these protective requirements, their environmental persistence has triggered an urgent shift toward biodegradable biopolymers [2]. However, a substantial performance gap restricts the commercial adoption of biopolymer films, which currently account for less than 1% of global plastic production [3]. Despite their rapid biodegradability and excellent gas-barrier properties, natural polymers suffer from low elongation at break (EAB) and poor water-vapour barrier performance [4,5,6,7]. Physical engineering strategies, including polymer blending, lamination, and emulsification, partially address these limitations but often introduce issues such as matrix delamination or require high plasticiser concentrations that compromise tensile strength (TS) [8].

To definitively resolve these macroscopic vulnerabilities, molecular-level structural modification via covalent cross-linking is essential [9]. Conventional physical treatments (e.g., thermal curing, irradiation) typically yield weak intermolecular networks [10], while synthetic chemical agents (e.g., glutaraldehyde, epichlorohydrin, formaldehyde) raise severe toxicological and regulatory concerns for food-contact applications [11,12,13,14]. Although natural cross-linkers like vanillin, gallic acid (GA), and citric acid offer less toxic alternatives [15,16], enzymatic cross-linking represents a highly sustainable and specific pathway; it operates under mild physiological conditions and avoids toxic synthetic reagents, yet the toxicological and migration profiles of its reaction by-products still demand rigorous safety assessment [17].

While enzymes such as transglutaminase, peroxidase, and tyrosinase have shown efficacy in biopolymer modification [18,19,20,21,22], laccase (*para*-benzenediol: oxygen oxidoreductase, E.C. 1.10.3.2) has emerged as a uniquely versatile biocatalyst [23]. Unlike glucose oxidase (GOx) (an oxygen scavenger) [24,25] or transglutaminase (a specific protein cross-linker) [26], laccase acts as a multifunctional catalyst [23,27,28]. It simultaneously mediates mechanical reinforcement, active headspace oxygen scavenging [29,30], and antimicrobial functionalisation across both proteinaceous (e.g., collagen, gelatine, whey protein) and polysaccharide-based (e.g., chitosan) matrices [27,28,31,32,33,34,35,36]. Despite early progress in laccase-mediated biocomposites [37,38] and recent advances in protein engineering and immobilisation [39,40,41,42,43,44], the existing literature remains highly descriptive and fragmented [45,46,47,48]. Recently, Alizadeh Sani et al. provided a valuable overview of laccase properties in food packaging [23]; however, significant knowledge gaps persist regarding the fundamental molecular mechanisms of interfacial coupling and the practical techno-economic feasibility of industrial scale-up.

To bridge the divide between laboratory prototypes and commercial implementation, this review systematically evaluates the biochemical and translational landscape of laccase-mediated packaging. First, we contrast the distinct kinetic and topological features of direct homopolymerisation versus mediator-assisted cross-linking. Second, we examine how diverse immobilisation architectures govern macroscopic mass-transfer regimes. Finally, we critically discuss the safety and toxicological profiles of reaction by-products, assess the techno-economic reality of enzyme production, and navigate the complex European and US regulatory frameworks, concluding with a forward-looking analysis of biomimetic nanozymes.

## 2. Laccase-Mediated Film Engineering: Mechanisms and Strategies

### 2.1. The Enzymatic Tool

Laccases are versatile multicopper oxidases whose widespread evolutionary distribution has been robustly mapped across diverse phylogenetic kingdoms. As demonstrated by comprehensive comparative genomics platforms screening hundreds of genomes, laccase and multicopper oxidase sequences are fundamentally conserved across fungi, bacteria, archaea, plants, and insects [49,50,51,52]. While these extensive phylogenomic analyses confirm that laccase-like domains are also sparsely present in other metazoan and vertebrate genomes [49,53], those found in mammals lack the canonical ability to oxidise phenolic substrates [54], rendering them unsuitable for biotechnological material design. Conversely, fungal laccases, particularly those secreted by white-rot basidiomycetes, have traditionally dominated industrial applications. Their widespread use is primarily attributed to their high redox potential at the T1 copper site (often exceeding +700 mV vs. NHE), which endows them with a superior thermodynamic capacity to initiate radical coupling across a broad range of food-safe aromatic target monomers [50,53,55]. Interestingly, the engineering goal of utilising laccase to mechanically reinforce biopolymer films finds a direct structural parallel in nature. In insects, laccases naturally drive cuticle sclerotisation, a biological process that profoundly hardens and waterproofs the exoskeleton through laccase-mediated protein cross-linking [52,53]. Translating this natural hardening paradigm into biomaterials science, materials scientists and packaging engineers can exploit laccase-mediated cross-linking to transform inherently fragile biopolymers into robust, water-resistant films that serve as sustainable alternatives to conventional plastics.

Laccases are monomeric, dimeric, or tetrameric glycoproteins classified within the multicopper oxidase family and the cupredoxin superfamily. As detailed by Singh and Gupta, their macromolecular architecture features a highly variable carbohydrate moiety, predominantly comprising mannose, *N*-acetylglucosamine, and galactose residues, which can account for 10% to 45% of the total protein mass [56]. This extensive glycosylation is not merely a structural by-product but a critical evolutionary feature that confers substantial baseline stability to the enzyme against proteolytic and environmental degradation [56]. Embedded within this robust glycoprotein framework, the catalytic core of each monomer incorporates four copper atoms distributed across three distinct sites: the Type 1 (T1) “blue” copper centre, the Type 2 (T2) “normal” copper centre, and the Type 3 (T3) coupled binuclear copper centre [50,56,57]. These enzymes exhibit broad substrate specificity, catalysing the oxidation of a diverse array of aromatic compounds, including phenols, anilines, aromatic thiols, some synthetic dyes, and polycyclic aromatic hydrocarbons [41,50,51,52,53]. The catalytic mechanism proceeds through three primary stages: the initial reduction of the T1 Cu^2+^ ion by the reducing substrate, subsequent internal electron transfer to the trinuclear copper cluster (TNCC) formed by the T2 and T3 sites, and the final reduction of molecular oxygen to water at the TNCC [41,42,58].

**Figure 1:**
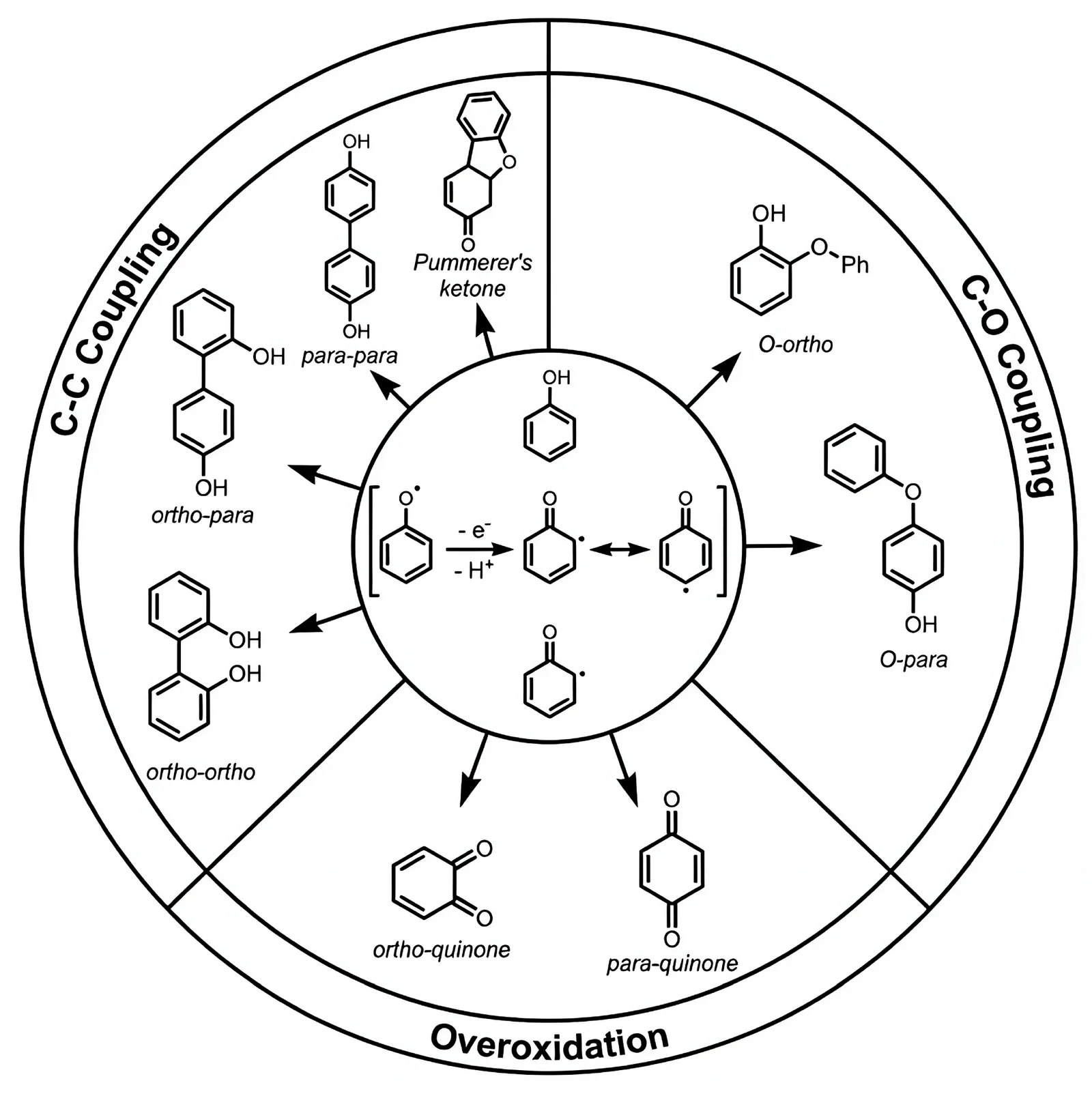
Mechanistic pathways of the aryloxy radical from laccase-catalysed phenol oxidation. The parent unsubstituted phenol is used as a simplified homotopic model (where *ortho* positions are equivalent), whereas in *meta*-substituted derivatives, these positions are constitutionally non-equivalent. Resonance-stabilised radical intermediates undergo regioselective coupling to yield: (i) C–C coupling products (*ortho*–*ortho*, *ortho*–*para*, and *para*–*para* dimers, and the tricyclic Pummerer’s ketone skeleton, which stably accumulates in *para*-substituted derivatives like *p*-cresol); (ii) C–O coupling products (*O*-*ortho* and *O*-*para* ethers); and (iii) overoxidation products (*ortho*- and *para*-quinones).

Figure 1 shows the divergent pathways available to an aryloxy radical generated *via* the one-electron oxidation of a monophenol by laccase. Fundamentally, the phenoxyl radical is resonance-stabilised, with the unpaired electron delocalised across the π-system [57,58]. As depicted in the central bracketed structures, this spin density is distributed specifically at the oxygen atom, as well as the *ortho* and *para* carbon positions of the aromatic ring. This delocalisation in the radical intermediate governs the regioselectivity of subsequent coupling reactions, leading to three primary reaction trajectories: C–C coupling, C–O coupling, and overoxidation [57,59]. In particular, the combination of two carbon-centred radicals leads to the formation of biphenyl dimers, which, depending on which resonance contributors participate, can be linked through *ortho*-*ortho*, *ortho*-*para*, or *para*-*para* linkages. Additionally, intramolecular rearrangement following coupling of *para*-substituted phenols, such as *p*-cresol, can yield tricyclic structures, such as Pummerer’s ketone [59]. Alternatively, coupling may occur between an oxygen-centred radical and a carbon-centred radical, generating ether linkages and producing either O-*ortho* or O-*para* adducts [60]. Crucially, the formation of these dimeric species does not necessarily represent the termination of the reaction. Since many of the resulting C–C and C–O coupled dimers retain phenolic hydroxyl groups (or gain reactive sites), they remain substrates for the enzyme. Consequently, they are susceptible to subsequent oxidative cycles and iterative radical coupling, processes that ultimately drive the formation of higher molecular weight (MW) oligomers and complex polymers [27,60,61].

**Figure 2:**
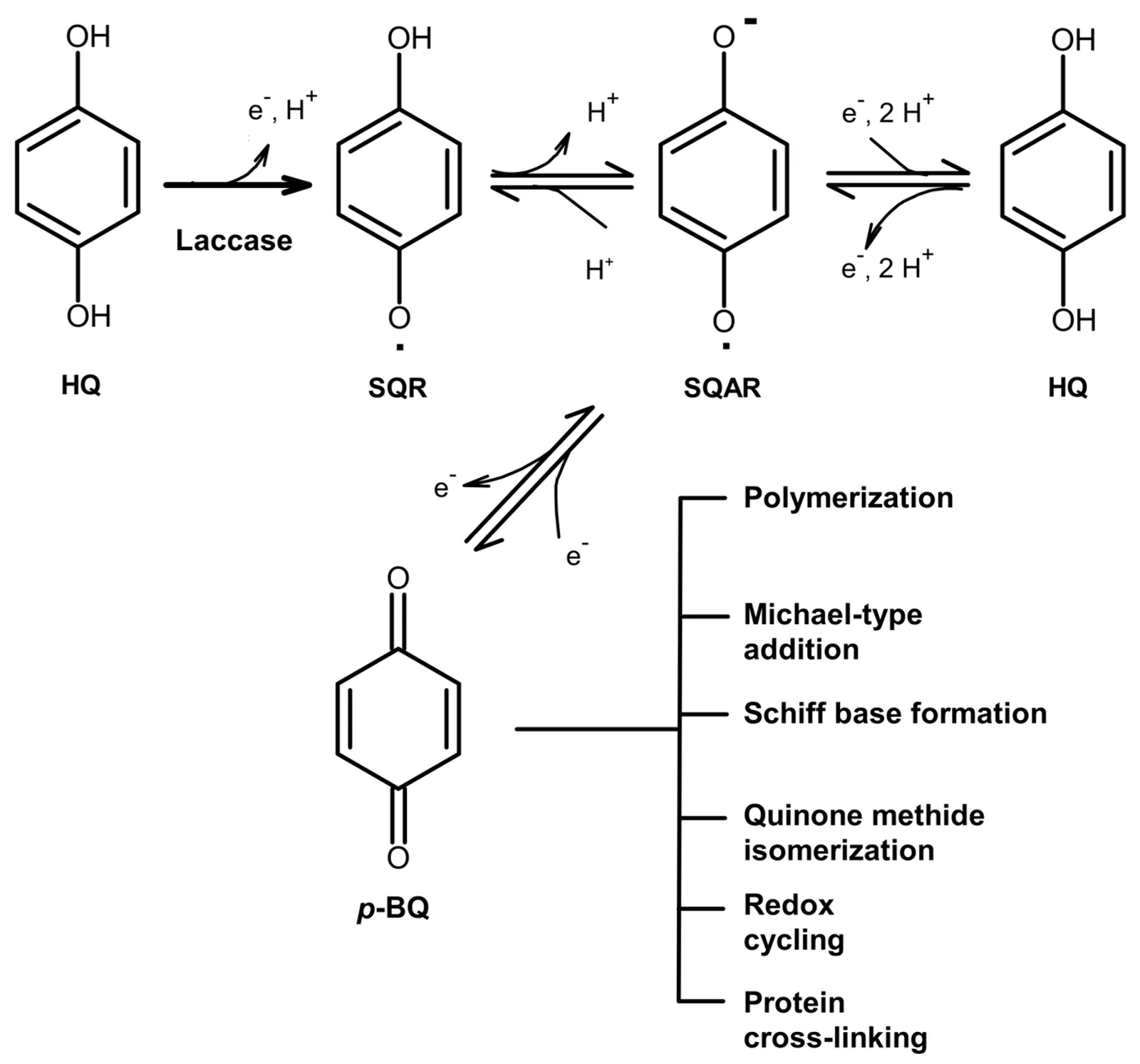
Reaction mechanism of laccase-mediated hydroquinone (HQ) oxidation and downstream electrophilic pathways of *para*-benzoquinone (*p*-BQ). The schematic highlights the transient radical intermediates (SQR and SQAR), the disproportionation-driven substrate recycling, and the diverse non-enzymatic covalent routes (e.g., Michael-type additions, Schiff-base formation, and protein cross-linking) governing biopolymer matrix reinforcement.

Regarding overoxidation products, several studies have reported the formation of either benzoquinones or quinone methides from the laccase-catalysed conversion of monophenols [62,63] or compounds bearing monophenol moieties [64]. However, the second oxidation step required to generate these products must compete with fast bimolecular radical coupling, which limits their formation. Conversely, benzoquinone formation during the laccase-catalysed oxidation of diphenols, such as hydroquinone (HQ; Figure 2), proceeds easily *via* a radical-mediated mechanism [65], owing to the significantly lower redox potential of diphenols compared to monophenols [55]. This thermodynamic property drives the disproportionation to benzoquinones rather than overoxidation. The reaction is initiated by the enzymatic abstraction of an electron and a proton from the hydroxyl moiety of HQ, generating the unstable semibenzoquinone radical (SQR), which upon deprotonation yields the semibenzoquinone anion radical (SQAR) [66]. Subsequently, two SQAR equivalents undergo disproportionation: one radical is reduced to regenerate the starting substrate (HQ), while the other is simultaneously oxidised to form *p*-benzoquinone [67].

This non-enzymatic mechanism is a critical feature of diphenol oxidation kinetics, ensuring continuous substrate recycling while accumulating the quinone product [68]. Far from being mere degradation end-products, quinones act as potent electrophilic agents susceptible to a variety of reactions [35,69,70,71] (Figure 2). Their presence is instrumental in the covalent cross-linking and structural tailoring of biopolymer-based films by leveraging the intrinsic nucleophilicity of macromolecular targets such as bovine serum albumin (BSA) [72], collagen [27], and chitosan [73].

Although wild-type laccases are widely employed, their structural vulnerability under harsh processing conditions often limits industrial scale-up. To overcome these barriers, recent advancements in protein engineering, specifically rational design and directed evolution, have facilitated the development of ‘tailored enzymes’ [42]. For instance, sequence saturation mutagenesis has generated *Melanocarpus albomyces* laccase variants exhibiting a threefold increase in catalytic turnover (*k*_cat_) at alkaline pH of 9.8 compared to the native enzyme [74]. Furthermore, in silico structural modifications targeting ionic interactions and disulphide bonds have successfully generated highly thermostable *Pleurotus ostreatus* laccase mutants, rendering these engineered biocatalysts significantly more robust against the demanding thermal and chemical stresses encountered in active-packaging manufacturing [75].

While traditional fungal laccases have dominated early research, their industrial implementation is frequently hindered by intrinsic biochemical limitations, notably a rapid loss of catalytic activity at temperatures exceeding 50 °C and at pH values above 6 [41,60,76]. To overcome these processing constraints, biopolymer engineering is increasingly shifting focus toward bacterial laccases, predominantly sourced from *Bacillus* and *Streptomyces* species [51]. These prokaryotic enzymes possess remarkable intrinsic robustness, demonstrating exceptional thermal stability, tolerance to high salt concentrations, and the ability to maintain significant catalytic efficiency across a much broader pH spectrum, often from neutral to alkaline [41,51]. For industrial scale-up, bacterial platforms offer decisive economic advantages over fungal systems; their rapid growth kinetics in submerged cultures and the absence of complex post-translational glycosylation requirements enable massive heterologous overexpression in hosts such as *Escherichia coli*, thereby drastically reducing fermentation times and production costs [43].

A highly successful practical validation of this alternative sourcing was provided by Zheng et al., who bypassed the inherent vulnerabilities of fungal laccase preparations by employing a recombinant, thermo-stable and pH-tolerant bacterial laccase from *Bacillus vallismortis* [77]. This robust bacterial biocatalyst was efficiently utilised to catalyse the functional grafting of GA onto chitosan, demonstrating that engineered non-fungal laccases are highly viable tools for scalable biopolymer modification [77]. Recent applications of robust prokaryotic enzymes, such as the ’small laccase’ (SLAC) from *Streptomyces coelicolor*, to stabilise chitosan–pectin blends revealed a highly unconventional mode of employment [78]. Given the fundamental absence of canonical oxidisable phenolic substrates within these matrices, conventional laccase-mediated covalent coupling was theoretically precluded. Instead of executing its copper-dependent catalytic cycle, the laccase protein itself paradoxically functioned as a physical polyelectrolyte bridging agent; its charged surface residues drove microstructural densification by mediating strong electrostatic complexation between the polycationic chitosan and polyanionic pectin, as evidenced by significant zeta-potential shifts [78]. Therefore, before delineating specific covalent cross-linking strategies, it is imperative to establish a rigorous distinction: future materials research must carefully decouple the true catalytic oxidation of laccases from their potential physical role as structural macro-ampholytes when applied to non-phenolic composite matrices.

### 2.2. Mechanisms of Laccase-Mediated Polymer Engineering

This section delineates the enzymatic modification of biopolymers *via* laccase-mediated cross-linking, offering a sustainable materials-engineering platform that aligns with the established green chemistry principles of in vitro enzymatic polymerisation [79]. By contrasting direct intrinsic cross-linking with mediator-assisted coupling pathways, we elucidate how these distinct chemical trajectories enhance the structural and functional properties of biopolymers, thereby providing advanced methods for macroscopic film design. Furthermore, surface-grafting approaches limited strictly to the functionalisation of biopolymers, without the formation of interchain covalent networks, are also critically analysed.

#### 2.2.1. Direct Homopolymerisation (Intrinsic Crosslinking)

**Figure 3:**
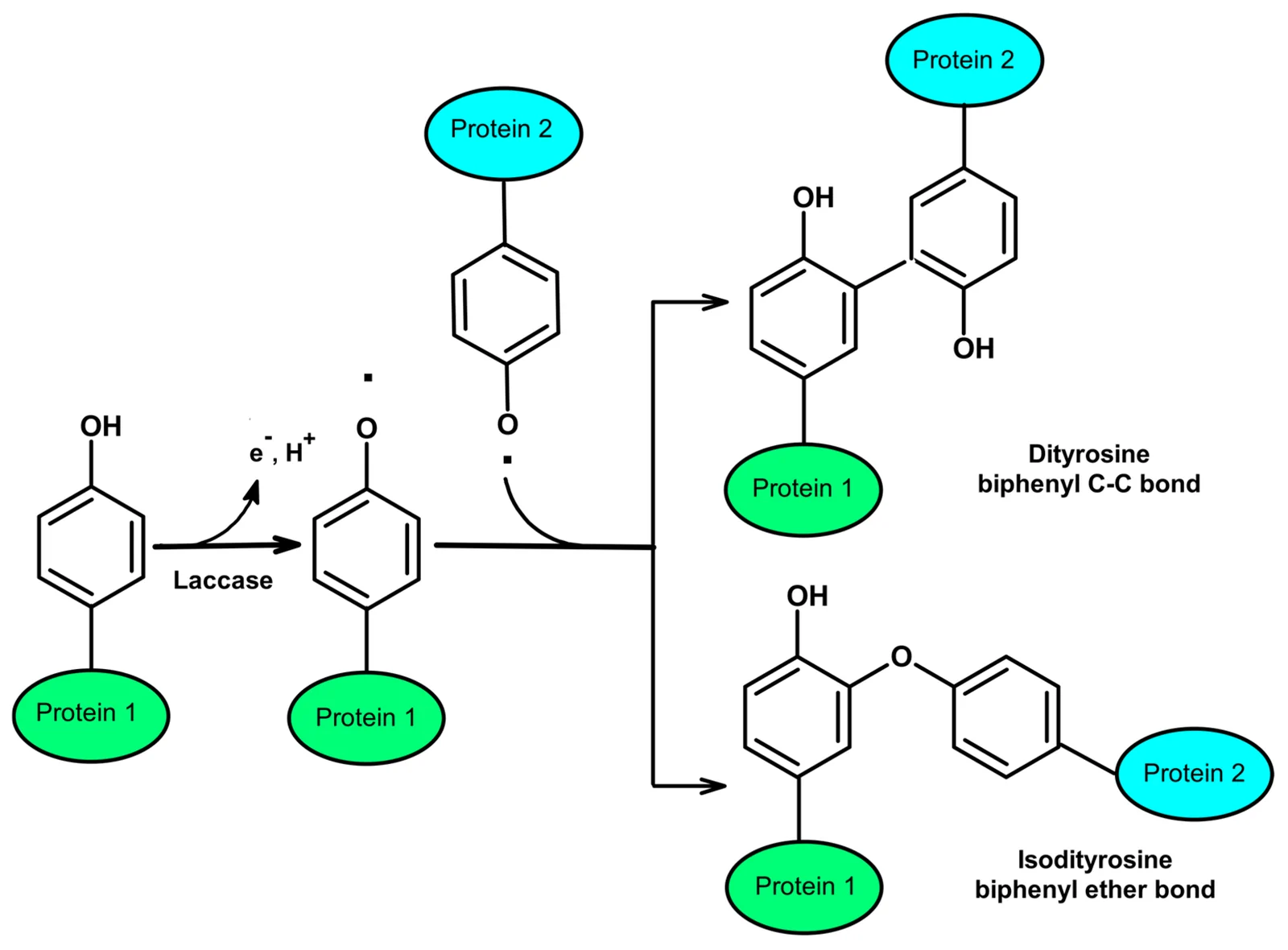
Schematic mechanism of laccase-catalysed direct homopolymerisation of proteins. One-electron oxidation of tyrosine residues leads to radical–radical coupling, yielding dityrosine (biphenyl C–C bond) and isodityrosine (diaryl ether C–O bond) cross-links.

Direct homopolymerisation by laccase is exclusive to proteins, as it relies on the oxidation of intrinsic phenolic amino acid residues, principally tyrosine, in the absence of exogenous phenolic mediators. As robustly mapped in the foundational literature on biocatalytic macromolecular synthesis, these enzymatic oxidative couplings govern the controlled propagation pathways that ultimately drive the formation of higher MW oligomers and complex polymeric networks [79]. The canonical mechanism is the one-electron oxidation of the phenolic ring of tyrosine to generate a tyrosyl radical, which undergoes radical–radical coupling to yield dityrosine or isodityrosine connected by a C–C biphenyl bond and a C–O diaryl ether bond, respectively [58,59,69,70,72], as shown in Figure 3. Tyrosine oxidation can also proceed *via* quinoid intermediates, with significant consequences for cross-linking, since quinones, due to their electrophilicity, can undergo 1,4-Michael addition with ε-amino groups of lysine, forming C–N linkages, or with other nucleophilic side chains (e.g., histidine, cysteine) when spatially accessible within the protein scaffold.

As highlighted by Heck et al., this cascade of spontaneous follow-up reactions renders laccase-driven homopolymerisation mechanistically highly diverse, generating a complex array of covalent cross-links that includes C–C, C–O, C–N, and potentially C–S bonds, depending on the spatial accessibility of other nucleophilic residues, such as cysteine [80].

However, for direct enzymatic cross-linking in food packaging applications, severe intrinsic limitations compromise macroscopic performance. Mattinen et al. established the ‘topological dogma’ of intrinsic cross-linking by demonstrating that laccase exclusively oxidises unshielded tyrosine, cysteine, and tryptophan residues [81]. Consequently, the enzyme failed to induce intermolecular cross-links in structured globular proteins (e.g., BSA and β-lactoglobulin), where these reactive sites are deeply buried within the hydrophobic core [81]. Subsequent studies on globular proteins [82] and macroscopic evaluations of biofilm-forming matrices such as collagen and gelatine confirmed that this strict steric restriction severely limits direct enzymatic reinforcement [69,70]. Combined with the extremely low natural abundance of exposed tyrosine (approximately 0.5%), direct enzymatic treatment often yields negligible dityrosine formation. Consequently, laccase alone frequently fails to provide meaningful mechanical reinforcement to the macroscopic film, occasionally even inducing competing partial peptide degradation rather than the desired polymerisation [69]. To overcome this steric barrier, early engineering approaches employed aggressive thermal denaturation to unfold the hydrophobic core and expose reactive tyrosine residues [83,84]. Nevertheless, forcing direct homopolymerisation with excessive laccase dosages created a critical trade-off: radical-induced peptide cleavage triggered protein fragmentation, thereby weakening the resulting macromolecular network [85].

This macroscopic failure is not limited to highly structured globular proteins. As demonstrated by Juvonen et al., attempts to cast films from intrinsically disordered proteins, such as sodium caseinate, using *Trametes hirsuta* laccase failed to yield an insoluble matrix [86]. Instead of forming a robust cross-linked network, the non-mediated enzymatic oxidation induced partial protein degradation and phase separation, leaving the resulting films highly susceptible to water solubilisation [86]. Furthermore, the laccase enzyme itself is a highly structured protein, and its surface-exposed residues may inadvertently undergo auto-immobilisation, irreversibly cross-linking into the newly formed polymer network and restricting its catalytic turnover [80].

To overcome these steric limitations, Quan et al. achieved highly efficient direct homopolymerisation of whey protein isolate (WPI) by employing a compact, two-domain bacterial ‘small laccase’ (SLAC) from *Streptomyces coelicolor* [76]. Following thermal unfolding of the protein target, the spatial accessibility of SLAC enabled direct tyrosine oxidation and dityrosine coupling without phenolic mediators, resulting in a 95% increase in tensile strength (TS) and outstanding UV-blocking properties [76]. However, a critical microstructural trade-off was evident: cross-sectional scanning electron microscopy (SEM) and dynamic light scattering (DLS) analyses revealed competing enzymatic cleavage of peptides and fragmentation of the protein matrix, leading to the accumulation of small degradation fragments (1–1000 nm) within the interstitial matrix. When reaction times exceeded the optimal threshold of 40 min, this competitive decomposition destabilised network cohesion, causing a notable decline in TS and a severe loss of film flexibility [76]. The robustness of this SLAC-driven architectural solution was subsequently corroborated in complex packaging systems; Zhou et al. demonstrated that SLAC successfully cross-links WPI even when blended with pectin, yielding composite films with significantly reduced water-vapour and oxygen permeabilities alongside enhanced TS [33]. Thus, although direct homopolymerisation is generally limited by steric constraints, the strategic combination of substrate thermal unfolding and compact bacterial biocatalysts, such as SLAC, represents a highly viable clean-label pathway for engineering robust proteinaceous packaging films [33,76].

#### 2.2.2. Functionalisation-Driven Crosslinking (Mediated/Grafting Strategy)

The historical paradigm shift toward mediated (or functionalisation-driven) cross-linking originated directly from the macroscopic mechanical failures of direct homopolymerisation. Early biochemical studies established that mediator-driven approaches could overcome the enzymatic inertia of tightly folded proteins. For instance, while transglutaminase is generally ineffective at cross-linking tightly folded globular proteins such as β-lactoglobulin, early foundational work demonstrated that adding ferulic acid (FA) to laccase generates small, highly mobile radical mediators capable of penetrating the globular core [82]. This radical-shuttling mechanism was rapidly extended to larger macroscopic structures by Cura et al., who demonstrated that integrating FA dramatically accelerated intermolecular cross-linking in sodium caseinate matrices, yielding significantly higher viscoelastic storage moduli than in unmodified gels [85]. Based on these structural foundations, Jus et al. translated this strategy into robust packaging architectures [69,70]. They demonstrated that adding small phenolic molecules (e.g., catechin or CA) to recalcitrant collagen and gelatine matrices allowed these mobile ‘bridging agents’ to bypass the steric hindrance of the protein core. Upon laccase oxidation, they covalently coupled to the sparsely available amino acid residues, driving a massive increase in the polymer’s MW and ultimately achieving the enhanced structural stability and TS that direct enzymatic treatment failed to deliver [69].

Qiang et al. furthered the strategy by providing definitive spectroscopic validation of this protein-bridging concept, using laccase-oxidised catechins to cross-link bovine collagen [87]. Using high-resolution X-ray photoelectron spectroscopy (XPS), the authors confirmed covalent Schiff base coupling *via* a drop in the surface O/C atomic ratio (from 0.89 to 0.50) and a threefold increase in the imine (=N–R) nitrogen peak at 398.79 eV (from 2.54% to 7.76%). This submicroscopic rearrangement hydrophobically densified the collagen fibrils, translating macroscopically into a 132% increase in tensile strength (to 9.33 MPa) and a significant reduction in the swelling ratio (from 273.82% to 169.91%), establishing a direct structure–property bridge essential for high-moisture packaging applications [87].

Expanding this concept to even larger polyphenols, Duan et al. incorporated gallotannins (GT), the simplest hydrolysable tannins, into acid-swollen collagen matrices [88]. While direct laccase treatment provided limited mechanical reinforcement, the laccase–GT mediator system generated an abundance of reactive quinones. These electrophilic intermediates reacted covalently with the nucleophilic residues of the collagen fibrils, yielding a highly compact laminar structure that significantly elevated both TS and thermal stability, thereby proving that mediator complexity directly dictates the macroscopic robustness of the protein network [88].

Engineering efforts extended this mediated strategy to polysaccharides (such as chitosan, starch, and cellulose), which inherently lack the functional groups required for direct enzymatic oxidation [48,89]. In this two-step ‘graft-then-link’ approach, small phenolic acids (e.g., FA or GA) serve as graftable anchor points. In the initial step, laccase oxidises the phenolic acid to generate a radical, which then reacts with a functional group on the biopolymer (typically a free amino group in chitosan), thereby grafting the phenolic ring onto the polymer backbone [73,90]. Laccase can then further oxidise these grafted tails, driving intermolecular coupling between adjacent polymer chains (e.g., through diferulic acid bridges) [45]. This strategy permits chemically inert polysaccharides to mimic the cross-linking behaviour of reactive proteins, delivering a twofold engineering advantage: exceptional structural reinforcement through high-density cross-linking and targeted antioxidant activity derived from unreacted grafted phenols [23,34].

**Figure 4:**
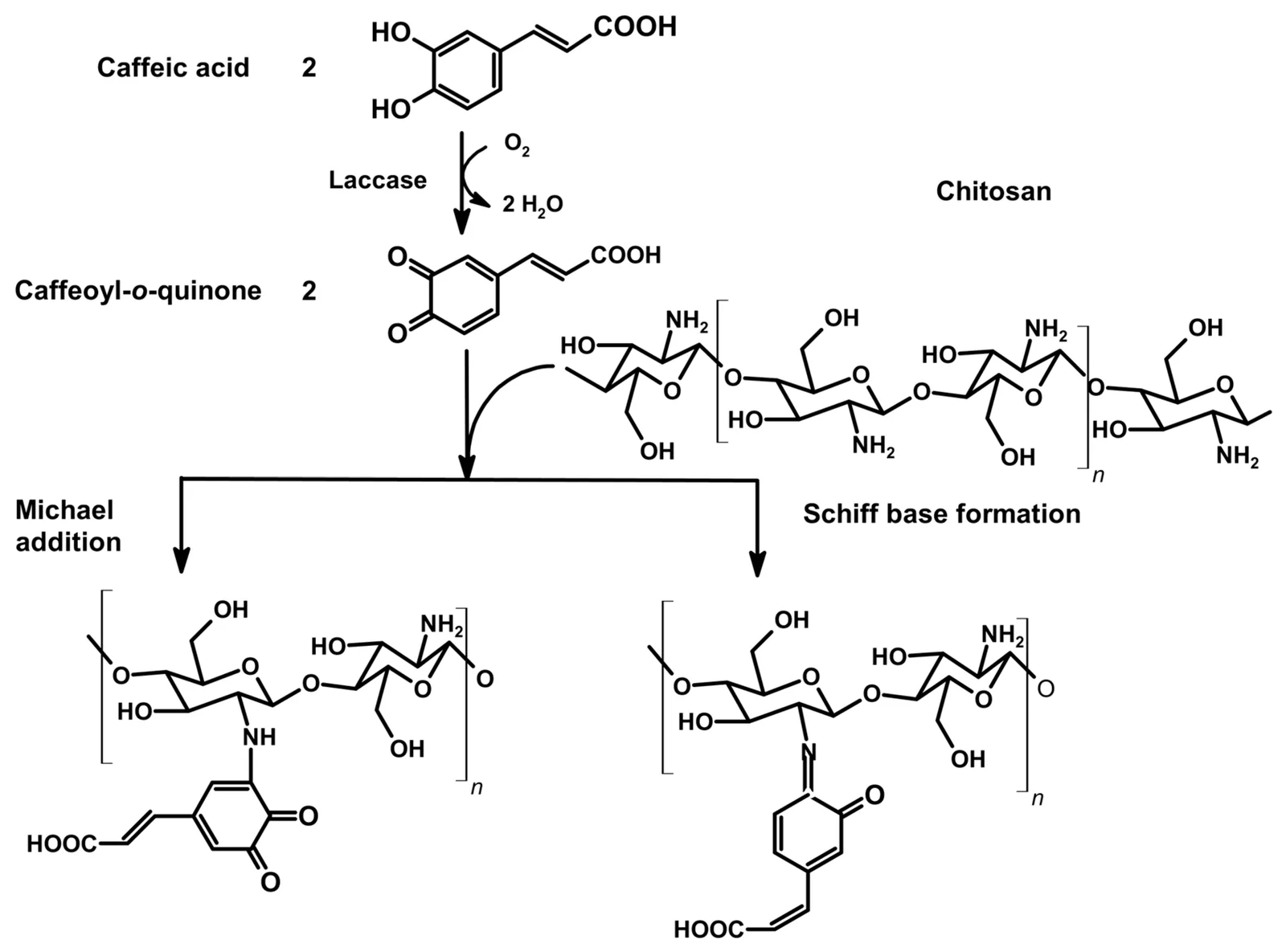
Reaction pathways of laccase-mediated functionalisation of chitosan with caffeic acid. At pH values preserving the nucleophilicity of chitosan’s amino groups, the enzymatically generated caffeoyl-*o*-quinone undergoes covalent grafting onto the chitosan backbone *via* Michael-type addition (**left**) and Schiff-base formation (**right**).

The pH of the reaction medium functions as a critical physicochemical switch, strictly governing the dominant grafting pathway by modulating the protonation state of chitosan. Božič et al. showed that the laccase-mediated functionalisation of chitosan with GA and caffeic acid is highly pH-dependent: in the 5.5–6.5 range, canonical Schiff-base and Michael-type addition reactions dominate, driven by the nucleophilicity of the polymer’s free amino groups [90]. This dual-pathway covalent coupling mechanism between the enzymatically generated caffeoyl-*o*-quinone and the nucleophilic amino group of chitosan is schematically illustrated in Figure 4. The transient in situ generation of such highly reactive *o*-quinonoid intermediates and their immediate susceptibility to nucleophilic amine additions represent a powerful chemical platform that has been successfully exploited even in advanced multi-enzymatic cascade configurations (e.g., compartmentalised laccase-lipase complexes) to drive the precise synthesis of structured alternating copolymers under green, mild buffered conditions [71].

However, at pH 4.5, where chitosan’s amino groups are fully protonated and thus non-nucleophilic, the covalent conjugation pathway shifts from amine-directed covalent addition to alternative interactions: covalent ester bond formation (for GA) or strong, non-covalent electrostatic complexation (for CA). This complete transition in reaction mechanisms demonstrates that pH modulation can be strategically engineered to precisely dictate the macromolecular linkage chemistry [90].

Furthermore, the molecular structure of the selected phenolic mediator dictates the optical and functional properties of the resulting bioplastic. Aljawish et al. reported that the laccase-catalysed oxidation of FA yielded a multitude of coloured oxidation products conferring a distinct yellow-orange hue and superior antioxidant activity on chitosan films [73]. Conversely, utilising its esterified analogue, ethyl ferulate, restricted the oxidation pathway, yielding completely colourless films with moderate radical-scavenging capacity. This highlights how the rational selection of the phenolic precursors’ side chains enables engineers to precisely tailor the packaging’s visual transparency and bioactivity to meet specific consumer acceptability standards [73].

Beyond the pre-selection of non-chromogenic mediators, Gonçalves et al. demonstrated that when highly effective but intensely chromogenic cross-linkers (such as genipin) are unavoidable, laccase can be strategically deployed not as a coupling agent but as an advanced post-processing bleaching tool [91]. While genipin severely restricts transparent packaging applications by imparting a dark bluish-green hue to chitosan matrices, treating the cured film with a recombinant bacterial laccase (CotA from *Bacillus subtilis*) and 2,2′-azino-bis(3-ethylbenzothiazoline-6-sulfonic acid) (ABTS), followed by a brief hydrogen peroxide step, successfully disrupts the highly conjugated systems responsible for the colouration [91].

This strategy successfully restores visual transparency while fully preserving the network’s acid stability and antioxidant capacity, thereby circumventing the degradative limitations of conventional chemical bleaching [91].

Furthermore, the specific covalent pathways of cross-linking are strictly governed by the MW and steric bulk of the phenolic mediator. While small phenolic acids readily undergo both Schiff-base and Michael-type addition reactions, the incorporation of large, complex polyphenolic extracts fundamentally shifts this scenario. As demonstrated by Chen et al. using ^13^C NMR analysis, the extreme steric hindrance of tea polyphenols completely precluded canonical Schiff-base condensation with chitosan [35]. Consequently, laccase-generated quinones reacted with the polymer’s primary amines exclusively *via* Michael-type addition. This underscores that the mediator’s architectural bulk serves as a selective thermodynamic switch, precisely dictating the final macromolecular linkage within the biopolymer network [35].

Functionalisation of aminated biopolymers relies on the Schiff-base or Michael-type addition reactions involving the amino (–NH_2_) groups. However, recent advances have extended the enzymatic toolbox to non-aminated polysaccharides. As a demonstration of laccase-mediated grafting, Zhang et al. successfully anchored FA on arabinoxylan (AX), a complex non-starch hemicellulose [92]. In the absence of amino nucleophiles, the reaction exploits the aliphatic hydroxyls of AX, specifically the primary C-5 hydroxyls of arabinofuranose side chains and secondary hydroxyls of the xylopyranose backbone, as oxygen nucleophiles [92]. Upon laccase-catalysed oxidation of FA into electrophilic quinonoid intermediates, the hydroxyl groups of AX undergo an *oxo*-Michael-type conjugate addition targeting the electron-deficient aromatic ring of the quinone. Detailed ^1^H and ^13^C NMR analyses indicated that the carboxyl group of FA remained intact, with covalent grafting proceeding exclusively *via* the aromatic ring to form stable C–O–C ether bonds [92]. This mechanistic breakthrough is significant because it demonstrates that laccase-mediated functionalisation is not limited to proteins or chitosan but can be engineered to work with a much broader range of plant-derived polysaccharides.

Expanding beyond the use of small-molecule mediators or free amino acids, recent advancements have demonstrated that laccase can directly couple entire polysaccharide chains to proteinaceous matrices. In a complex ternary blend of sericin, gelatine, and carboxymethyl chitosan (CCS), Ma et al. employed laccase to selectively oxidise the intrinsic tyrosine residues of the proteins into *o*-quinonoid intermediates [93]. These reactive intermediates subsequently underwent spontaneous Schiff-base condensation and Michael-type addition reactions with the primary amino groups of the CCS chains. This advanced macrografting approach essentially eliminates the need for small-molecule mediators, relying instead on interpolymeric covalent coupling to yield a highly entangled, structurally dense composite network.

#### 2.2.3. Surface Functionalisation (Grafting Without Network Formation)

Unlike cross-linking, surface functionalisation strategies are employed when the primary objective is to impart specific bioactivities (e.g., antioxidant or antimicrobial activity) or physicochemical traits (e.g., hydrophobicity) without inducing rigid inter-chain coupling. Based on the pH-dependent thermodynamic principles elucidated by Božič et al., conducting the enzymatic reaction under acidic conditions (e.g., pH 4.5) ensures that the amino groups of chitosan remain fully protonated and protected from canonical Schiff-base cross-linking [90].

Exploiting this chemical environment, Liu et al. utilised laccase to graft 4-hexyloxyphenol (HP), a bulky monophenol, onto the chitosan backbone [34]. While this functionalisation imparted exceptional antioxidant capacity and significantly increased the surface hydrophobicity (elevating the water contact angle to 70°), the study revealed a critical engineering trade-off. The introduction of these bulky hydrophobic side chains physically disrupted the native inter- and intramolecular hydrogen bonding within the chitosan matrix, thereby decreasing polymer crystallinity. Consequently, the engineered copolymer exhibited a simultaneous decrease in both TS and EAB compared to unmodified chitosan, demonstrating that heavy grafting of bulky monophenols inherently induces matrix weakening, compromising the macroscopic film integrity [34].

Recognising these mechanical limitations, subsequent engineering efforts shifted focus from freestanding solid films to supported architectures, where the functionalised biopolymer forms a coating or supporting layer rather than shouldering the primary structural load. This progression led to the transition to liquid edible coatings, an architectural shift systematically established in 2018 by parallel seminal studies that relied on recombinant bacterial laccases to synthesise highly bioactive liquid preservatives. While Zheng et al. utilised this approach to graft GA onto chitosan for meat preservation [77], Yang et al. simultaneously proved its effectiveness on agricultural commodities by grafting FA onto chitosan [94]. By applying this enzymatic conjugate as a liquid spray coating on fresh mangoes, Yang et al. sidestepped the need for macroscopic structural rigidity. Instead, they relied on the coating’s fluid dynamics to form a uniform, barrier-enhancing layer on the fruit’s cuticle [94].

In parallel with the development of these fluid bio-interfaces, a second major architectural pathway in surface functionalisation has focused on solid porous supports. The integration of cellulosic substrates to support enzymatically active interfaces is rooted in the pioneering laccase antibacterial surface process (LASP) developed by Elegir et al. [37]. In this foundational approach, laccase was utilised to directly graft phenolic compounds, such as CA and isoeugenol, onto unbleached Kraft liner fibres, specifically exploiting the residual surface lignin as a covalent anchoring platform to achieve robust bactericidal activity against *S. aureus*. To advance supported biopolymer architectures, Ni et al. bypassed the mechanical trade-off associated with the bulky HP mediator by coating Kraft paper with a laccase-HP-chitosan conjugate [95]. While the underlying cellulose fibres ensured dry structural integrity, the grafted copolymer formed a continuous hydrophobic shield. Although HP’s steric bulk predictably hindered interfacial hydrogen bonding, this water-resistant barrier effectively protected internal cellulosic networks from moisture penetration, elevating the sizing degree from 0.27 to >330 s and driving a sixfold increase in wet TS [95]. Collectively, these advancements demonstrate that the mechanical vulnerabilities of heavily grafted biopolymers can be smoothly circumvented by deploying them as supported surface films, either directly on food matrices or on supportive cellulosic substrates.

### 2.3. Immobilisation Architectures: Kinetics and Performance Implications

The functional efficacy of laccase-engineered films depends not only on the enzyme’s catalytic potential but also on its spatial arrangement within the polymeric matrix. Although recent reviews have documented the stabilising effects of immobilisation, particularly its role in preventing enzyme leakage and enhancing thermal durability [23], there is limited critical analysis of the kinetic implications associated with different immobilisation architectures. The distinction between bulk entrapment, where the enzyme is buried within the matrix core, and surface immobilisation, where the enzyme is interfacially exposed, determines the material’s mass-transfer regime and, consequently, its suitability for specific packaging applications.

#### 2.3.1. Entrapment and Casting (Matrix Burial)

While physical entrapment represents the most conceptually straightforward immobilisation strategy, the direct embedding of native laccases within polymeric networks frequently exposes the biocatalyst to severe operational vulnerabilities. Early proof-of-concept studies, such as the foundational work by Chatterjee et al., attempted to directly entrap laccases within latex/clay coatings [96]; however, residual enzyme activity plummeted to 18–53% strictly due to the thermal and dehydrating stresses associated with the film-forming process. Furthermore, even when extreme thermal curing is avoided, conventional bulk entrapment fails to prevent long-term thermodynamic destabilisation. As demonstrated by Jhuang et al., laccase immobilised within standard, non-electrospun biopolymer films lost over 40% of its initial catalytic activity after only 10 days of refrigerated storage [97,98]. This rapid inactivation, driven by unfavourable microenvironmental changes and the progressive loss of the enzyme’s essential hydration shell, explains why the simple physical entrapment of unprotected native enzymes has largely been bypassed in modern packaging design.

To definitively address this critical microenvironmental vulnerability, recent engineering paradigms have shifted towards advanced protection strategies. One approach utilises natural deep eutectic solvents (NADES) to form robust supramolecular networks that serve as artificial hydration shells, thereby drastically enhancing the enzyme’s thermal stability under severe heating [39,89].

Another strategy relies on hierarchical compartmentalisation: Wang et al. demonstrated that microencapsulating laccase within highly porous sodium alginate/soluble starch microcapsules shields the enzyme from thermal denaturation while selectively permitting substrate diffusion into the bulk hydrogel [99]. Beyond native proteins, the integration of biomimetic nanozymes offers a radical solution for processing stability [100]. Bio-inspired copper MOF nanozymes (e.g., Cu^I^_0.6_Cu^II^_2.4_-MOF) emulate the laccase active site while exhibiting exceptional thermal resilience, retaining over 80% of their activity even after direct exposure to 90 °C [101] and maintaining catalytic integrity across wide temperature ranges and during long-term storage [102].

Crucially, once the biocatalyst is adequately stabilised through these advanced formulations or biomimetic strategies, the intrinsic mass-transfer and kinetic limitations of bulk entrapment, which typically restrict substrate diffusion and reduce apparent enzyme activity due to steric constraints on active sites, shift from a processing penalty to distinct materials-engineering and regulatory advantages [23,89]. While burying the catalyst slows down rapid surface reactions, this exact architecture is optimal for oxygen scavenging [29,30]. From a regulatory perspective, deep matrix entrapment or covalent chemical anchoring is critical to attenuate any migration of the laccase macromolecule or its reactive intermediates into food simulants, thereby supporting compliance with non-migrating active packaging criteria (e.g., Regulation (EC) No 450/2009) [103]. Physically, the dense polymer network provides a protective shield that enhances laccase stability under the severe thermal and shear stresses encountered during film casting or coating [23]. Economically and operationally, for active preservation applications such as headspace oxygen-scavenging, this diffusion-limited regime is strategically beneficial; rather than triggering rapid catalytic cycles that would prematurely exhaust the sacrificial active compounds, the tortuous diffusion pathways within the solid film allow a slow, steady consumption of permeating oxygen [29,30]. This continuous transverse interception, triggered by moisture-induced polymer plasticisation (e.g., ≥92% RH), effectively neutralises gas permeation before it reaches the food matrix, establishing engineered bulk entrapment as a highly effective architectural design for long-term oxidative preservation [29].

#### 2.3.2. Surface Immobilisation (Nanofibre and Film Decoration)

To circumvent the severe mass-transfer limitations inherent to bulk entrapment, an alternative architectural paradigm involves immobilising laccase on the surface of pre-formed structures. By directly exposing the biocatalyst to the target environment, this interfacial architecture shifts the overall kinetics from diffusion-limited to a highly efficient, kinetically controlled regime. The foundational principles underpinning the superiority of this approach were demonstrated in early generic biocatalytic models that leveraged the exceptional surface-to-volume ratio (typically 100–1000 m^2^/g) of electrospun nanofibres [104].

Nonetheless, conventional two-dimensional (2D) surface immobilisation (e.g., direct adsorption or chemical grafting onto pre-formed flat films) is constrained by major thermodynamic and biochemical bottlenecks. Physical surface adsorption is inherently prone to sustained enzyme desorption under high-moisture conditions, compromising long-term sensor stability and raising safety concerns regarding food-contact migration [105]. Conversely, chemical surface modification *via* covalent anchoring successfully prevents desorption but often suffers from unproductive random enzyme orientation and overcrowding, which block active site channels and reduce apparent catalytic efficiency [60]. To overcome these challenges, active and intelligent packaging research has focused on three-dimensional (3D) electrospun polymer nanofibres. Thermodynamically, electrospinning drives solvent evaporation solely *via* a high-voltage electric field; this non-thermal process completely circumvents the heat-induced denaturation associated with conventional high-temperature solvent casting, ensuring maximal baseline laccase activity [106]. Topologically, the high specific surface area of electrospun networks (such as 11.58 m^2^/g for zein and 17.05 m^2^/g for chitosan) widely disperses the covalently bound enzyme molecules [97,98]. This high dispersion effectively prevents catalytic crowding and ensures that a dominant fraction of active sites remains sterically accessible to diffusing substrates, mitigating the kinetic penalty of random orientation [107,108]. The functional superiority of this nanofibrous surface-exposed architecture has been quantified across diverse biopolymers; Jhuang et al. demonstrated that transitioning from a standard 2D cast film to a 3D electrospun chitosan network increased relative catalytic activity more than fourfold (from 16.9% to 70.7%) while retaining over 94% of initial activity after 10 days [97]. Similarly, on electrospun zein fibres, the surge in specific surface area mitigated diffusion constraints so effectively that the system achieved 92.8% relative catalytic activity [98]. This maximised catalytic efficiency and unhindered access to active sites are essential for intelligent sensing applications, such as time-temperature indicators (TTIs), which strictly require rapid colourimetric kinetics in response to environmental fluctuations [107,108].

As a complementary nanostructured alternative to organic polymer fibres, hybrid inorganic–organic systems have emerged to protect surface-bound enzymes. Specifically, laccase can be encapsulated within ultrasonically synthesised copper phosphate hybrid nanoflowers [44]. This robust, high-surface-area architecture confers exceptional conformational rigidity, yielding a catalytic activity more than four times that of the free laccase while retaining approximately 92% of its initial activity after 30 days of storage [44].

## 3. Functional Performance of Laccase-Treated Materials

Laccase has gained significant traction in food packaging as a sustainable, safe alternative to petroleum-derived plastics and synthetic additives [23]. By oxidising renewable phenolic feedstocks, laccase-mediated fabrication strongly aligns with consumer demands for eco-friendly solutions [33,94,109,110,111]. Beyond its green profile, laccase catalysis fundamentally enhances functional performance by establishing robust moisture and oxygen barriers, scavenging residual headspace oxygen to prevent microbial and oxidative spoilage, and preserving the packaging matrix’s biodegradability, to mitigate end-of-life plastic accumulation [35,60,96]. To provide a systematic overview, Table 1 correlates recent laccase-engineered polymer–mediator combinations with their respective functional outcomes, demonstrating how biocatalysis concurrently imparts mechanical robustness, active preservation, and intelligent monitoring capabilities.

### 3.1. Mechanical and Barrier Reinforcement

Fundamentally, the macroscopic reinforcement of laccase-engineered biopolymers is governed by the covalent densification of the matrix. The magnitude of this mechanical and barrier enhancement is strictly dictated by the cross-linking density, which, in mediator-assisted systems, depends entirely on the molecular architecture and concentration of the selected phenolic agent [27,31,88]. Establishing this principle, Tang et al. demonstrated that among various phenolic acids, GA, possessing three available hydroxyl groups, maximised the generation of reactive quinones [27].

This led to extensive Schiff-base and Michael-type addition cross-linking within collagen films, generating a highly compact microstructural network that exceptionally increased TS to 147.65 MPa while significantly reducing water vapour permeability [27]. In a subsequent study, the same authors found that increasing the GA dosage further tightened the network, elevating the thermal denaturation temperature by 17.9 °C and imparting robust UV-shielding properties [31]. As demonstrated by Yin et al., the laccase-catalysed cross-linking of collagen with delphinidin yielded a composite matrix (Col/Dp–LA) that increased wet TS by over 13 MPa compared to the native polymer, providing the essential structural integrity required for high-moisture food environments [28].

Beyond simple phenolic acids, engineering efforts have successfully scaled this densification strategy to encompass complex polyphenols, enabling the macroscopic rescue of physically disrupted matrices. Duan et al. systematically demonstrated that integrating large amounts of GT into acid-swollen collagen films *via* laccase oxidation yielded a highly compact laminar structure with substantially elevated TS and thermal stability (109.07 °C) [88]. This structural reinforcement was synergistically amplified by coupling hydrolysed GT with nanosized, highly interactive pre-formed collagen fibrils, thereby producing bioplastics compatible with harsh thermal processing [112]. This exceptional mediator-driven densification also provides the structural basis for incorporating mechanically disruptive active phases, such as essential oil Pickering emulsions (PEs). While PEs typically plasticise and weaken biopolymer networks, Ran et al. showed that coupling cinnamon essential oil PEs with a laccase-oxidised mulberry extract generated a dense covalent network that fully counteracted the physical disruption, thereby restoring TS to values exceeding 106 MPa and significantly improving the moisture barrier [111].

In addition to mechanical reinforcement, mediator-driven cross-linking fundamentally alters surface wettability and diffusion pathways. The restriction of polymer segmental mobility directly governs this improvement in water resistance. Laccase-catalysed covalent cross-linking, proceeding *via* Schiff-base condensation and Michael-type addition, significantly densifies the three-dimensional network, reducing macromolecular free volume and constraining cooperative chain motions, as reflected in elevated glass transition temperatures (*T_g_*) and reduced swelling ratios [30,45]. By limiting polymer relaxation, this rigid network prevents water molecules from extensively swelling and plasticising the matrix, forcing them to navigate highly tortuous diffusion pathways rather than diffusing freely [30,45]. For example, Winestrand et al. reported that laccase-catalysed cross-linking of starch with fractionated lignosulphonates increased the polymer’s average MW and triggered a dramatic shift from a highly hydrophilic state to a hydrophobic one, achieving water contact angles up to 81° [30]. Shah et al. demonstrated that bacterial laccase acted as a targeted ‘depolymerisation tool’ prior to film casting [32]; by enzymatically degrading bulky alkali lignin into lower-molecular-weight fractions before blending with keratin, they achieved exceptional matrix homogeneity. This specific breakdown drastically reduced interstitial cavities, driving a substantial drop in WVP from 9.1 to 6.1 × 10^−10^ g·m^−1^·s^−1^·Pa^−1^ [32].

Enhancing barrier and mechanical properties through covalent cross-linking introduces a fundamental engineering trade-off, as the resulting restriction in polymer chain mobility significantly compromises material flexibility. Studies across various biopolymer matrices, such as FA-grafted chitosan [73] and highly cross-linked protein networks [87], demonstrated that increased structural rigidity inherently led to a substantial reduction in EAB. This macroscopic compromise, consistently observed in other polymers including pectin [113], dictates that researchers must carefully balance molecular rigidity with the macroscopic flexibility essential for the industrial machinability of packaging films. To resolve the mechanical rigidity–flexibility dichotomy, recent engineering strategies have employed advanced architectures and strictly controlled enzyme kinetics. For instance, utilising *S. coelicolor* laccase (SLAC) as a physical polyelectrolyte in chitosan–pectin blends simultaneously improved gas-barrier properties, TS, and EAB without requiring the covalent attachment of rigid aromatic moieties [78]. Another study pointed out that, when covalent reinforcement is applied, laccase concentrations must be rigorously optimised; exceeding optimal biocatalyst load leads to hyper-aggregation and localised over-cross-linking, restricting polymer chain mobility and severely compromising the material’s industrial heat-sealing performance [93].

### 3.2. Antioxidant and Antimicrobial Activity

Active packaging strategies leveraging laccase-mediated functionalisation offer a natural alternative to synthetic preservatives, though they entail a specific molecular trade-off. As demonstrated by Yin et al., the enzymatic consumption of free phenolic hydroxyl groups to generate cross-linking quinones slightly reduced the radical-scavenging capacity of the resulting matrix; despite this marginal reduction, covalent tethering remains vastly superior to simple physical blending [28]. Chen et al. showed that anchoring tea polyphenols to the chitosan backbone *via* laccase prevented the rapid leaching typically observed during processing, thereby ensuring a significantly higher retained antioxidant capacity [35]. Furthermore, this structural immobilisation fundamentally alters release kinetics; Lu et al. found that covalent linkages in pectin-based films prevent uncontrolled ‘burst releases’ [113]. By directly anchoring phenolics to the pectin backbone, the matrix establishes a sustained ‘contact-active’ mechanism that successfully inhibits microbial proliferation on highly perishable foods, such as shrimp, throughout a 7-day storage period [113].

Moreover, the ultimate functional profile of the network is strictly dictated by the specific molecular architecture and MW of the selected mediator, often presenting a distinct dichotomy between antioxidant and antimicrobial optimisation. For maximal radical-scavenging, minimising steric hindrance around the phenolic groups is important. Shah et al. demonstrated that actively depolymerising bulky lignin into low-MW fractions substantially increased the concentration of exposed hydroxyl groups, thereby providing significantly stronger DPPH radical-scavenging activity than native lignin [32]. Conversely, as reported by Athanasiou et al., prolonged enzymatic polymerisation of phenolic extracts resulted in severe steric hindrance, structurally shielding these groups and thereby critically compromising antioxidant capacity [61].

However, the opposite principle often applies to antimicrobial efficacy. As elucidated by Tian et al., while hydrolysed GT maximised mechanical density, intact GT exhibited superior antimicrobial efficacy due to their potent iron-chelating capacity, which deprives surface microbiota of essential growth elements [112]. Similarly, Elegir et al. established that laccase-generated oligomers exerted exponentially higher lethality than free monomers due to their highly branched structures and enhanced electron delocalisation [37]. Furthermore, the chemical nature of the monomer strictly governs this balance: Tang et al. observed that while GA optimised mechanical cross-linking, FA yielded the most potent antimicrobial activity (>97% inhibition against *E. coli* and *S. aureus*) [27].

A unique macromolecular trade-off emerges when the biopolymer matrix itself possesses intrinsic bioactivity, as in chitosan. The natural antibacterial efficacy of chitosan relies heavily on its positively charged free amino groups, which electrostatically disrupt bacterial cell membranes. Božič et al. demonstrated that cross-linking at pH 6.5 covalently consumed these critical amines *via* quinone coupling, thereby reducing the film’s intrinsic lethality [90]. Executing the functionalisation at pH 4.5 circumvented this vulnerability by preserving the protonated amino groups while grafting the phenolic acid *via* alternative esterification pathways, yielding a material with high antimicrobial and radical-scavenging properties [90]. Alternatively, this inherent amino group depletion can be circumvented *via* a ‘compensation mechanism’. As established by Itzincab-Mejía et al., rationally selecting a mediator with exceptionally high intrinsic antimicrobial potency, such as octyl gallate, successfully compensated for the reduction in polycationic charge, ultimately enhancing the macroscopic lethality against foodborne pathogens [114].

Mechanistically, functionalised networks exert a dual, synergistic antimicrobial effect: grafted phenolics disrupt bacterial membranes, causing cytoplasmic leakage [115], while the densified cross-linked matrix acts as a steric barrier that physically asphyxiates spoilage organisms by restricting gas diffusion [92]. The practical efficacy of these active systems has been validated across various perishable foods, ranging from liquid coatings that delay mango respiration [94] to solid films that inhibit lipid oxidation in minced beef [31]. Furthermore, to overcome the thermal instability of natural enzymes, recent advancements have integrated robust biomimetic nanozymes, such as copper-based metal–organic frameworks (Cu-MOFs) [100,116]. By replicating the multicopper active site of natural laccases, these nanomaterials structurally reinforce the biopolymer matrix while simultaneously conferring exceptional radical-scavenging and bactericidal properties, thereby ensuring the optimal preservation of highly perishable commodities [100,116].

### 3.3. Oxygen Scavenging Films

To prevent microbial and oxidative spoilage, active packaging relies on the removal of residual headspace oxygen. While iron-based scavengers dominate the commercial market, they pose significant industrial limitations, including triggering metal detectors, incompatibility with microwave heating, and generating metallic off-flavours [117]. Enzyme-based alternatives overcome these physical issues; however, the commonly used GOx generates cytotoxic hydrogen peroxide, strictly necessitating the co-immobilisation of catalase for neutralisation [118]. In contrast, laccase provides a cleaner and more straightforward scavenging mechanism by coupling the oxidation of phenolic substrates directly to the reduction of molecular oxygen into harmless water [23,29,96]. Crucially, this biocatalytic packaging is fully microwave-compatible. Unlike conventional oxygen scavengers based on zero-valent iron powders, which contain bulk metallic lattices that trigger inline metal detectors and cause electromagnetic arcing hazards [117], laccase-based films do not interfere with microwave radiation. Although laccase is a multicopper oxidase containing four catalytic copper ions per monomer [41], these transition metals are atomically dispersed and coordinatively bound within the glycoprotein scaffold [23]. Lacking a free-electron conduction band, the macromolecular matrix behaves electromagnetically as a non-conductive organic material, preventing any risk of shielding or localised overheating. While the high temperatures reached during microwave heating (typically >70 °C) denature and catalytically inactivate the laccase protein, this occurs solely at the point of consumption, long after the active barrier has completed its primary preservation function during storage. Whereas intense microwave heating triggers a rapid degradation and subsequent migration of unbound, synthetic antioxidants in conventional plastics [119], the covalent anchoring of both laccase and its phenolic substrates within the biopolymer network permanently confines these macromolecules, thereby substantially minimising the risk of heat-induced leaching into food matrices [28,113]. In addition to generating safer by-products, laccase systems demonstrate superior thermodynamic performance in cold-chain packaging, retaining over 20% of their room-temperature activity at 7 °C [96]. Furthermore, unlike GOx, which depends on hydrophilic glucose that diffuses poorly within hydrophobic polymers, the broad substrate promiscuity of fungal laccases enables the selection of moderately lipophilic phenolic scavengers [23,89]. This versatility effectively overcomes severe diffusion limitations, ensuring optimal mass transfer and kinetic efficiency within the solid packaging matrix.

Despite this substrate mobility, the macroscopic scavenging performance is ultimately governed by the matrix’s structural dynamics. The fundamental engineering principles for these active networks, established by Johansson et al., revealed a counterintuitive paradigm: the oxygen-scavenging capacity does not correlate linearly with the absolute concentration of free phenolic hydroxyl groups in the substrate [29]. Instead, continuous enzymatic oxidation within solid coatings requires a high-humidity trigger (≥92% RH) to generate sufficient molecular mobility through polymer plasticisation [29]. Advancing this concept chronologically, Winestrand et al. further optimised these laccase-catalysed networks by utilising fractionated industrial lignosulphonates [30]. Because these specific derivatives plasticise more readily, the authors successfully broadened the system’s operational window, achieving maximal scavenging efficiency at a significantly lower relative humidity threshold (≥84% RH) [30]. Ultimately, this inherent kinetic reliance on moisture dictates the commercial application of these functionalised films, making them exceptionally well-suited for preserving high-water-activity (*a*_w_ > 0.9) perishable commodities, such as fresh-cut fruits, meats, and cheeses, where the package’s internal microclimate naturally triggers and sustains the enzymatic oxygen consumption [29,30].

### 3.4. Intelligent Monitoring: TTIs and Direct Freshness Indicators

Intelligent packaging utilises colourimetric strategies for direct monitoring of spoilage metabolites and indirect thermal tracking via TTIs. Laccase-crosslinked matrices serve as excellent platforms for integrating natural pH-sensitive dyes. For example, physically doping a laccase–delphinidin–collagen film with *Vaccinium oxycoccus* pigment yields a purplish-red to greyish-blue transition in response to total volatile basic nitrogen accumulation in packaged fish [28]. Beyond physical incorporation, laccase was used to oxidise mulberry extract, generating reactive *o*-quinones that simultaneously cross-linked the collagen matrix and served as covalently embedded sensors [111]. This dual-function approach established a fully integrated active-and-intelligent system that shifted from red to blackish-green upon fish spoilage, eliminating the need for secondary indicator dyes [111].

Alternatively, TTIs provide real-time data on temperature abuse, offering a more reliable assessment of food deterioration than static expiration dates. However, their efficacy relies on predictive synchronisation: the activation energy (*E_a_*) of the TTI must closely match the food’s specific deterioration kinetics, typically within a margin of 25 kJ/mol [120,121]. Unmodified laccase TTIs often exhibit overly rapid kinetics leading to premature colouration, but they can be strategically modulated. Park et al. pioneered the use of sodium azide (NaN_3_), a non-competitive inhibitor that binds to the enzyme’s copper centres, to artificially expand the system’s *E_a_* from 48 up to 110 kJ/mol [122]. Translating this liquid-phase modulation to solid-state architectures, engineers have incorporated NaN_3_ into electrospun matrices. This induces a crucial ‘latency phase’ (chromatic hysteresis) that delays the onset of colour change, effectively preventing premature activation [108]. Jhuang et al., by jointly optimising laccase loading and NaN_3_ concentration, precisely tuned the *E_a_* of nanostructured TTIs to 67.9 kJ/mol on chitosan [97] and 76.97 kJ/mol on zein fibres [98] (Table 2).

This precise calibration enables the polymer reaction kinetics to perfectly superimpose on specific bacterial growth curves [122], significantly reducing prediction errors to below 3% for fresh-cut papaya spoilage [123].

To overcome the logistical hurdle of premature “automatic activation” immediately following fabrication, enzymatic TTIs require stabilisation strategies, such as thermodynamic suspension (e.g., freezing laccase-ABTS TTIs at −18 °C until on-demand thawing) [124] or mechanical compartmentalisation, to keep the enzyme and substrate physically separated prior to packaging [125].

The most advanced engineering paradigm couples conditional mechanical activation with biomimetic nanomaterials to concurrently bypass this premature activation and the thermal instability of natural enzymes. For instance, a highly thermostable copper-based nanozyme (Cu-BDC-NH_2_) was integrated into a ‘squeeze-type’ device and stored in a dormant state under a nitrogen atmosphere; activation occurred only when the user manually ruptured a micro-blister to introduce the O_2_ required for catalysis [102] (Table 2). Ultimately, substituting labile natural laccases with robust nanozymes yields an exceptionally stable, user-controlled colourimetric platform [101,102]. However, while these synthetic mimics (e.g., Cu-MOFs) offer superior thermal resilience, their actual food-contact applicability remains strictly constrained by unresolved nanosafety concerns [100]. Unlike naturally safe glycoproteins, the high risk of transition metal migration and ligand leaching poses severe toxicological and bioaccumulation hazards, necessitating separate, stringent pre-market safety evaluations within current active packaging frameworks (see Section 4.1.4 for a detailed safety discussion).

## 4. Critical Analysis of Industrial Viability (The “Gap” Section)

Despite notable advancements, the commercialisation of laccase-mediated food packaging remains hindered by significant technical and economic barriers. As outlined by Venkatesan et al., the general industrial scale-up of biopolymer films is constrained by poor machinability, high production costs, and the complex migration kinetics of active additives [9]. For laccase-engineered systems, these macroscopic challenges translate into critical knowledge gaps regarding the toxicological safety of enzymatic reaction by-products and the overall techno-economic viability of large-scale biocatalytic processing.

### 4.1. Safety and Migration Risks

#### 4.1.1. Toxicological Profile of Laccases and Active Additives

A primary safety consideration in laccase-mediated food packaging involves the toxicological profile of the enzyme itself and the residues remaining in the polymer matrix after processing. The oral toxicity of fungal laccases from non-genetically modified wild-types has been recently subjected to rigorous evaluation by the European Food Safety Authority (EFSA) [126]. Evaluating a laccase from *Trametes hirsuta* (strain AE-OR), the Panel established a No Observed Adverse Effect Level (NOAEL) of 862 mg Total Organic Solids (TOS)/kg body weight (bw) per day. Based on a conservative dietary exposure model, the Margin of Exposure (MoE) was calculated to exceed 28,000, concluding that the enzyme does not pose safety concerns under intended conditions of use. For recombinant laccases (e.g., *Myceliophthora thermophila* expressed in *Aspergillus oryzae*), systematic toxicological assessments conducted under the guidelines of the Joint FAO/WHO Expert Committee on Food Additives (JECFA) confirmed the absolute absence of genotoxic, mutagenic, or subchronic oral toxicity [127]. JECFA-compliant manufacturing processes further guarantee that commercial recombinant laccase preparations (such as the commercial formulation *Flavourstar*) are completely free of fungal mycotoxins (including cyclopiazonic acid, kojic acid, and 3-nitropropionic acid) below strict detection limits [128]. However, powdered enzymes present occupational inhalation risks (respiratory sensitisation) [129,130] during dry handling. To mitigate these hazards, international standards recommend transitioning to liquid or dust-free granular co-formulations [131]. Within the completed packaging, covalent immobilisation, which permanently confines the enzyme molecules, aims to restrict their migration into food simulants, thereby facilitating compliance while safeguarding the packaging’s thermal and catalytic stability [27]. In this context, simple phenolic substrates frequently selected for film engineering, such as GA, FA and CA, are structurally classified under Cramer Class I (representing low-toxicity monocyclic structures), corresponding to a high safe human exposure threshold of 1800 µg/person/day [132,133]. These dietary phenolics benefit from well-established metabolic clearance pathways, and several compounds, such as vanillin, hold Generally Recognised as Safe (GRAS) status under US FDA regulations, including Title 21 of the Code of Federal Regulations (CFR) §182 [134]. Conversely, more complex polyphenols such as catechin are classified under Cramer Class III due to their benzopyran core, which triggers a far more restrictive safety threshold of 90 µg/person/day [132,133]. This structural dichotomy highlights why achieving highly efficient covalent cross-linking is critical for complex polyphenols; permanently confining these macromolecules within the polymeric matrix is critical to minimise their potential migration into food simulants, thereby facilitating compliance with strict regulatory safety margins [27,90].

#### 4.1.2. Coupling Efficiency and o-Quinone Reactivity

The biocatalytic cycle of laccase relies on the one-electron oxidation of phenolic hydrogen donors, generating highly reactive phenoxyl radicals and quinone intermediates [50]. A critical toxicological concern is whether these reactive electrophilic species can migrate into food simulants and cause cellular damage. However, the current literature indicates that when laccase is used to functionalise biopolymer films, the reactive intermediates are quantitatively consumed *via* rapid, irreversible covalent coupling (such as Schiff-base condensation or Michael-type addition) with the amino groups of chitosan or protein matrices [90].

This high coupling efficiency has been shown to substantially mitigate the cytotoxicity associated with free phenolic monomers. For instance, laccase-mediated grafting of GA onto chitosan networks not only preserves but enhances the biocompatibility of the film, maintaining over 80% cell viability in L929 fibroblasts at concentrations up to 1000 µg/mL [115]. Similarly, laccase-cross-linked collagen films functionalised with GA or GT demonstrated a total absence of in vitro cytotoxicity against L929 fibroblasts, proving that the covalent entrapment of the oxidised phenolics in the insoluble proteinaceous network is exceptionally stable and safe for food-contact applications [27,112].

#### 4.1.3. Threshold of Toxicological Concern (TTC) and Migration Modelling

When specific toxicological data for complex, newly synthesised reaction products or for unreacted low-molecular-weight phenolic precursors acting as non-intentionally added substances (NIAS) are lacking, the Threshold of Toxicological Concern (TTC) framework, recommended by the EFSA Scientific Committee, serves as an invaluable predictive screening tool [132]. Under this established decision tree, safety limits are strictly dictated by chemical structure and genotoxic potential [132]. Specifically, migrating compounds structurally identified and classified under Cramer Class I (representing simple, low-toxicity structures) are assigned a safe human intake threshold of 30 µg/kg body weight (bw) per day, whereas complex phenolics and oligomeric coupling products typical of Cramer Class III are restricted to a much lower threshold of 1.5 µg/kg bw/day [132,133]. Crucially, if in silico (Q)SAR modelling or in vitro assays (e.g., Ames test) reveal potential DNA-reactive mutagenicity alerts for reactive quinone intermediates, the safety threshold drops drastically to 0.0025 µg/kg bw/day, equivalent to 0.15 µg/person/day) [132]. For completely unknown or structurally uncharacterised NIAS detected during chromatographic screening, a highly conservative worst-case threshold of 0.15 µg/person/day must be applied to comprehensively exclude mutagenic risks [135,136]. To systematically evaluate whether potential migration levels exceed these TTC limits, deterministic mathematical migration models based on Fick’s second law of diffusion are routinely utilised [137]. By incorporating the diffusion coefficient (*D*) of the migrant in the biopolymer, the partition coefficient (*K*) at the packaging–food interface, and the thermal exposure profiles during storage, these models predict real-time exposure [137,138]. Crucially, while ‘Safe-by-Design’ covalent immobilisation provides an outstanding chemical barrier to restrict migrant mobility, compliance with TTC thresholds cannot be assumed solely from structural design. Instead, safety validation mandates a synergistic approach combining experimental migration assays (e.g., chromatographic screening *via* HPLC-MS/MS in food simulants) with validated, case-specific mathematical diffusion modelling to rigorously verify chemical safety prior to commercialisation [135,136,137].

#### 4.1.4. Safety Bottlenecks in Food Nanozymology

While laccase-mimicking nanozymes, such as Cu-MOFs, offer extraordinary advantages in thermal stability and lower manufacturing costs, they introduce critical nanosafety and toxicological bottlenecks absent in native, biologically derived laccase glycoproteins [100]. Because Cu-MOFs consist of synthetic, crystalline coordination networks, there is a persistent risk of hydrolytic collapse of the framework, which can drive the subsequent leaching of free copper ions, residual organic ligands, or intact MOF nanoparticles into food matrices.

Once migrated, these species induce cytotoxicity through the intracellular generation of Reactive Oxygen Species (ROS) *via* Fenton-like and Haber–Weiss-type pathways, leading to oxidative stress, membrane damage, and potential genotoxicity upon long-term exposure [100,101]. Under current European regulations, the use of engineered nanomaterials in food contact materials (FCMs) is subject to extremely strict pre-market approval requirements. Regulation (EC) No 450/2009 explicitly states that the authorisation for an active substance does not automatically cover its nanoform; hence, separate, dedicated nanosafety assessments (evaluating particle-specific migration, cellular uptake, and long-term bioaccumulation) must be performed for Cu-MOFs, establishing a formidable regulatory and industrial barrier compared to laccases [100,139].

### 4.2. Techno-Economic Reality: Cost of Enzyme vs. Value of Food Saved

From a macroeconomic perspective, the global laccase enzyme market is experiencing rapid expansion, valued at USD 1.2 billion in 2025 and projected to reach USD 2.1 billion by 2034 with a compound annual growth rate (CAGR) of 6.8% [140]. Food and beverage applications represent a highly significant segment, accounting for 18.2% of global consumption (approximately USD 218.4 million in 2025) [140]. Commercial market availability is highly consolidated, with major global manufacturers, such as Novonesis, DuPont/IFF, Amano Enzyme Inc., Yiduoli, Sunson, and Denykem, dominating the industrial supply chain and controlling approximately 98% of the global market share [41]. Fungal laccases remain the standard platform, representing 45.0% of the market share, with industrial production primarily relying on high-cell-density submerged fermentation (SmF) [140]. To meet strict industrial and food-contact requirements, manufacturers standardise the purity of these commercial enzyme preparations to ensure homogeneous specific activity. For instance, commercial food-grade liquid formulations (such as *Flavourstar*) are characterised by a TOS content standardised around 5.3% *w*/*w* [128], ensuring highly consistent catalytic performance and predictable mass-transfer kinetics within the film-forming process.

To systematically evaluate the industrial scalability of enzyme production and biopolymer film engineering, this review adopts the Technology Readiness Level (TRL) framework, which maps technology development from basic laboratory proof-of-concept (TRL 3) to competitive commercial manufacturing (TRL 9). Originally developed for aerospace systems, the TRL scale has been successfully adapted to chemical and process engineering to assess both the reduction in technical uncertainty and the economic viability of novel bioprocesses at desired production volumes [141]. Within this framework, a quantitative techno-economic analysis is imperative to demonstrate whether implementing active enzyme-mediated layers can balance production costs with measurable shelf-life extension benefits [105].

Upstream, the economic feasibility of laccase-based technologies is strictly determined by the production platform and downstream purification costs, systematically compared in Table 3. Although commercial laccase preparations have been utilised industrially since the mid-1990s, their market prices exhibit extreme variability (EUR 0.34 to >130/kU) depending on specific activity and purity requirements [40,41]. Furthermore, because most bulk commercial preparations are tailored for the textile or environmental sectors, they lack the food-grade certifications required for direct food contact [89]. This shortfall forces packaging facilities to either incur the high overheads of stringent purification or develop localised, food-safe fermentation strategies [41].

Historically, conventional submerged fermentation (SmF) has presented high operational expenditures (OPEX), with synthetic culture media accounting for up to 89% of the total production cost [142]. Localised solid-state fermentation (SSF) of agro-industrial by-products reduces raw material expenses to 31–33% of the total OPEX [143], achieving a minimum laccase selling price (MLSP) as low as USD 0.05/kU [144]. Alternatively, high-density heterologous expression in *Pichia pastoris* utilising biodiesel-byproduct glycerol as a cost-effective carbon source significantly enhances volumetric productivity, limiting unit production costs to EUR 0.34/kU [40]. Because downstream purification remains a primary cost driver, utilising crude or partially purified ultrafiltration extracts represents the most pragmatic approach for bulk active films [146].

Downstream formulation and deployment strategies must minimise both the cost of mediators and the absolute enzyme payload required per packaging unit. Circular economy initiatives demonstrate that unrefined agricultural wastes, such as wine lees, can successfully substitute expensive synthetic mediators to yield active films [61]. Simultaneously, deploying laccase onto high-specific-surface-area carriers, such as electrospun nanofibres, drastically minimises the required enzyme payload per sensor [107], a configuration further optimised by using robust laccase-inorganic hybrid nanoflowers [44]. To circumvent the cost bottlenecks associated with natural enzymes, biomimetic nanozymes represent a highly competitive alternative; the synthesis of a bio-inspired trinuclear copper organic framework reduces production costs by 16.2-fold relative to equivalent amounts of natural laccase, while offering a 37.2-fold increase in catalytic activity [101].

Ultimately, the techno-economic viability of these architectures depends on a compelling cost–benefit balance, in which the cost of enzyme-mediated film fabrication must be offset by reduced food spoilage during storage and distribution. Crucially, a clear distinction must be made between the pure enzyme-dosing cost and the total film-fabrication cost. Based on an active packaging dosage of 0.1 to 1.0 U/cm^2^ and a minimum laccase selling price (MLSP) ranging from EUR 0.05/kU (*via* solid-state fermentation of agricultural wastes) to EUR 0.34/kU (*via* heterologous *Pichia pastoris* platforms), the pure enzyme dosing cost is extremely low, ranging from EUR 0.01 to 0.10 per m^2^ of film. In contrast, the total film fabrication cost, including raw biopolymer carriers (e.g., chitosan or whey protein at EUR 2.00–5.00/kg), plasticisers (e.g., glycerol), solvents, and processing utilities (e.g., extrusion or solvent casting), is estimated to range from EUR 0.80 to EUR 2.50 per m^2^, depending on film thickness (30–100 µm) and processing methodology. Consequently, the active biocatalyst represents only a minor fraction (from 2% to 10%) of total material and manufacturing costs. When translating these metrics to packaged goods, a standard pouch or tray-wrap configuration for 1 kg of a high-protein commodity (e.g., fresh minced beef or fish fillets) requires an estimated packaging surface area of approximately 0.10 m^2^ (based on a standard 15 cm × 25 cm wrapped footprint). Applying these spatial parameters to 1 kg of packed food, the enzyme dosing cost is merely EUR 0.001–0.01, while the total film fabrication cost ranges from EUR 0.08 to 0.25.

Highly perishable, high-protein food commodities, such as minced beef and fresh fish, are prone to rapid deterioration, leading to retail and consumer waste rates of up to 50% [147]. By establishing robust active and intelligent barriers, laccase-mediated systems can reduce this waste to 2.5% [28,31]. Given the wholesale prices of high-protein foods, this net waste reduction prevents economic losses exceeding EUR 2.00 per kilogram of packed food [147]. Thus, the economic value of the food saved exceeds the pure biocatalytic dosing cost by two orders of magnitude and comfortably offsets the total film fabrication cost by a factor of 8 to 25.

The technology readiness levels, representative systems, and technical bottlenecks of the primary active and intelligent laccase-mediated packaging configurations are comprehensively summarised in Table 4.

**Table 4:** Techno-economic and Technology Readiness Level (TRL) evaluation of laccase-mediated packaging systems.

Packaging Application (TRL) ^‡^	Representative System	Economic & Deployment Metrics	Primary Technical Bottlenecks	Key References
Active Antimicrobial/Antioxidant Films (TRL: 3–4)	Direct/grafted chitosan or protein matrices (e.g., GA/FA-chitosan, collagen-GT).	• Dosing cost: Estimated at EUR 0.01–0.10 per m^2^ of film. • Value saved: Reduces spoilage of high-protein foods (beef, fish); retail waste can drop from ~50% to 2.5%, saving > EUR 2.00/kg.	• Rigid network decreases film flexibility (low EAB). • Localised over-crosslinking compromises heat-sealing. • Migration risk of reactive quinones and unreacted low-MW species (NIAS).	[27,31,37,48,88,90]
Active Headspace Oxygen Scavengers (TRL: 4)	Bulk-entrapped laccase in coatings (e.g., starch-lignosulfonates).	• Dosing cost: Low-cost utilising agri-food lignosulphonates as sacrificial substrates. • Value saved: High cold-chain stability (retains > 20% activity at 7 °C); avoids co-immobilized catalase.	• Absolute reliance on high humidity (RH ≥ 84–92%) for activation. • Thermal inactivation risks during thermoplastic casting.	[29,30,96]
Intelligent Time-Temperature Indicators (TRL: 4–5)	Surface-immobilised laccase on nanofibres or hydrogel microcapsules.	• Dosing cost: Low payload due to high specific surface area of nanofibres (up to 11.58 m^2^/g). • Value saved: Colourimetric cold-chain monitoring; tunable apparent *E_a_* (26 to 110 kJ/mol) matched to food.	• Risks of premature activation, needing frozen storage (−18 °C) or mechanical separators before use. • Use of toxic inhibitors (NaN_3_) raises safety issues.	[97,98,107,123,124]
Nanozyme-Based Indicators & Active Layers (TRL: 3)	Biomimetic copper networks (e.g., Cu-MOFs, Cu-BDC).	• Dosing cost: Synthesis costs are 16.2-fold lower compared to natural laccase. • Value saved: Retains > 80% activity at 90 °C; 37.2-fold activity increase; zero protein denaturing risks.	• Safety and toxicity profiles of synthetic nanostructures are not yet fully standardised. • Strict premarket approval required for food contact.	[100,101,139]

^‡^ Technology Readiness Levels (TRLs) are defined according to the EU Horizon 2020 framework; see Table 3 footnote for detailed scale criteria.

### 4.3. Regulatory Status: Specific Hurdles for Enzymatically Active Packaging

The commercial translation of laccase-based active packaging is hampered by a complex regulatory landscape under both European and US jurisdictions. In the European Union, food-contact materials (FCMs) must comply with the general safety requirements of the Framework Regulation (EC) No 1935/2004, ensuring that the transfer of constituents does not pose a risk to human health. While laccase as a processing aid is regulated under Regulation (EC) No 1332/2008, active enzyme residues incorporated into packaging films to interact with the food or headspace cannot benefit from this classification. Instead, they fall under the strict scope of Regulation (EC) No 450/2009 for active and intelligent materials, which explicitly excludes active components from the ‘functional barrier’ exemption, thereby requiring explicit pre-market authorisation based on EFSA safety dossiers [103]. Similarly, the US FDA adopts a dual-pathway classification based on the active agent’s release mechanism: substances migrating to exert a technological effect within the food require pre-market approval as direct food additives under 21 CFR, whereas contact-active, non-releasing components are governed under the more streamlined Food-Contact Substance (FCS) notification program [135].

The ‘migration–reaction nexus’ represents the primary safety bottleneck: manufacturers must demonstrate that the in situ generation of reactive radicals and quinones does not lead to the migration of hazardous reaction products or uncharacterised NIAS into the food simulant. Any migrating reaction product or low-molecular-weight oligomer must comply with the specific migration limits (SML) and overall migration limits (OML) established by Regulation (EU) No 10/2011 for plastic layers [136]. Although the ‘Safe-by-Design’ covalent tethering of laccase and phenolic substrates aims to minimise heat- and moisture-induced leaching, compliance with specific (SML) and overall migration limits (OML) under Regulation (EU) No 10/2011 cannot be assumed solely from structural design [135,136]. Consequently, formal regulatory approval requires empirical migration testing with liquid food simulants, coupled with chromatographic screening (e.g., HPLC-MS/MS) and deterministic Fickian diffusion modelling, to confirm compliance under real-world shelf-life conditions. Furthermore, these regulatory hurdles are exacerbated by the introduction of biomimetic nanozymes, as both the FDA and EU frameworks require separate, stringent pre-market nanosafety evaluations for nanomaterials, making standardised protocols essential for commercial viability [138].

## 5. Conclusions and Future Outlook

Laccase-mediated food packaging has successfully transitioned from a theoretical biocatalytic concept to a robust material engineering platform capable of bridging the performance gap of biopolymer-based plastics. While laboratory-scale formulations have demonstrated outstanding functional efficacy in mechanical and barrier properties, the next evolutionary step toward commercial viability demands a critical shift from batch processing to high-throughput industrial manufacturing. Since conventional thermal extrusion or melt-blending easily denatures the laccase protein scaffold, integrating low-temperature wet-coating technologies, such as slot-die or industrial spray-coating, onto pre-extruded sustainable carriers (e.g., polylactic acid or paperboard) represents a viable alternative for preserving enzymatic activity in continuous Roll-to-Roll (R2R) production lines.

In parallel, maintaining enzyme stability under operational shear and dehydration during extended storage remains essential. This challenge can be effectively addressed by shifting from free enzymes to high-surface-area immobilisation on food-grade inorganic carriers (such as bentonite or halloysite) or within electrospun polymer nanofibres, which physically shield the protein structure. Alternatively, the deployment of copper-based metal–organic frameworks as biomimetic, laccase-like nanozymes offers an exceptionally stable and cost-effective substitute, reducing raw material costs while displaying robust resistance to thermal and physical processing stresses.

However, the commercial application of MOFs for direct food contact is currently constrained by critical and unresolved consumer safety concerns. The potential migration of copper ions and residual organic ligands from the crystalline MOF scaffold into food matrices represents a major toxicological hurdle, posing risks of bioaccumulation, systemic toxicity, and cellular oxidative stress that remain insufficiently characterised under actual shelf-life conditions. Consequently, establishing rigorous nanosafety protocols and conducting exhaustive migration kinetics studies in food simulants are prerequisites for assessing their real-world biocompatibility and ensuring compliance with European Food Safety Authority (EFSA) and Food and Drug Administration (FDA) safety standards.

Beyond these nanostructured alternatives, the industrial translation of natural enzymatic systems is intrinsically tied to the ‘Safe-by-Design’ paradigm, which mandates the elimination of chemical hazards at the earliest stages of formulation development. Rather than utilising synthetic and potentially harmful mediators, future developments must rely exclusively on natural, food-grade phenolic compounds, such as CA, FA, or GA, covalently grafted onto the biopolymer backbone to substantially minimise their release into the packaged food. This covalent tethering approach, applied to both the mediators and the laccase macromolecule itself (e.g., onto chitosan), is designed to restrict migration to levels well below the TTC limits, thereby facilitating regulatory clearance through standardised migration assays.

## Figures and Tables

**Table 1:** Overview of laccase-engineered biopolymer films: cross-linking strategies, active agents, and macroscopic functional outcomes.

Biopolymer Matrix	Mediator/Active Agent	Primary Functional Enhancements	References
Collagen	Gallic acid	Improved tensile strength and thermal stability; strong UV blocking, DPPH radical scavenging, and antimicrobial activity against *S. aureus* and *E. coli*.	[31]
Collagen	Mulberry extract	Simultaneous cross-linking and integration of built-in pH sensors; visual colour shift (red to blackish-green) indicating fish spoilage.	[111]
Collagen	Delphinidin	Increased wet TS; provided pH-responsive visual indication for total volatile basic nitrogen accumulation.	[28]
Chitosan	Tea polyphenols	Significantly enhanced TS and antioxidant capacity; reduction in EAB	[35]
Keratin	Alkali-lignin	Increased TS (14.8 MPa) and EAB (23.7%); enhanced thermal stability; suppressed proliferation of *S. aureus* and *E. coli*.	[32]
Whey Protein Isolate	None (Intrinsic crosslinking)	Structural densification leading to improved TS and decreased water vapour permeability.	[76]
Sericin/gelatine and Carboxymethyl chitosan	None (Heteropolymerisation)	Development of edible “cookable” packaging with controlled thermal disintegration in boiling water; outstanding antioxidant performance.	[93]

**Table 2:** Kinetic and Thermodynamic Calibration of Laccase-Based Time-Temperature Indicators (TTIs).

TTI Architecture & Carrier Matrix	Enzyme/ Substrate/ Inhibitor System	TTI Activation Energy (kJ/mol)	Target Food Matrix & Spoilage	Predictive Accuracy/Validation Outcome	References
Liquid phase model (Buffer solution)	Laccase/ABTS/ NaN_3_ (2.4 μM)	Tunable from 48.0 to 110.0 (Customised at 75.0)	*Pseudomonas fragi* growth in liquid model (75.0 kJ/mol)	Accurate synchronisation; consistent prediction of quality loss regardless of thermal history.	[122]
Electrospun nanofibres (Zein polymer)	Laccase/ Guaiacol/ NaN_3_ (100 μM)	26.28 (without inhibitor); up to 76.97 (with NaN_3_)	General Wide applicability	Excellent kinetic correlation (*R*^2^ = 0.995 for NaN_3_ formulation); hysteresis observed at low temperatures.	[98]
Electrospun nanofibres (Chitosan/PVA)	Laccase/ Guaiacol/ NaN_3_ (10 μM)	5.8–40.9 (without inhibitor); up to 67.9 (with NaN_3_)	General/Fresh-cut fruits and vegetables	High kinetic correlation (*R*^2^ > 0.88); thermal stability retained during cycling.	[97]
Electrospun nanofibres (Chitosan/PVA/ Si)	Laccase (20 μg/cm^2^)/ Guaiacol/ NaN_3_ (100 μM)	55.69	Fresh-cut papaya; microbial spoilage kinetics (70.49 kJ/mol)	Isothermal prediction errors < 2.78% (Avg: 1.50%); matched dynamic fluctuations.	[123]
Hydrogel microcapsules (Alginate/Starch in PVA/CMC)	Laccase/Guaiacol (Inhibitor-free; tuned *via* enzyme loading)	Tunable from 27.32 to 61.13 (Optimal TTI#1: 49.65)	*Agaricus bisporus* mushrooms (50.01 kJ/mol)	Δ*E_a_* = 0.36 kJ/mol; highly accurate dynamic response with an effective temperature difference (ΔT_eff_) of only 1.77 K under variable thermal cycles	[99]
Squeeze-Type nanozyme (Cu-BDC-NH_2_ MOF)	Laccase-like nanozyme/Guaiacol/O_2_-activated	Tunable from 28.45 to 72.85	Broad range (applicable to foods with 3.45–97.8 kJ/mol)	>90% accuracy post-activation; solves premature reaction via mechanical O_2_ release.	[102]

**Table 3:** Quantitative comparison of laccase production platforms and commercial viability for food contact, categorised by Technology Readiness Levels (TRLs).

Production Platform (TRL) ^‡^	Sourcing & Purity Status	Estimated Cost	Industrial Trade-Offs	Key References
**Submerged Fermentation** (**SmF**) (**TRL: 9**)	• Fungal strains (including wild-types, mutagenized hyper-producers, or homologous overexpressing strains) • Lacks food-grade certification (textile target).	EUR 0.34 to >130/kU (highly variable based on purification).	**(+)** Mature global market; stable multi-ton supply chains. **(−)** Prohibitive OPEX; synthetic culture media accounts for up to 89% of SmF total production costs.	[40,41,142]
**Solid-State Fermentation** (**SSF**) (**TRL: 5–6**)	• Fungal wild-types on agricultural residues. • Low-to-medium purity; typically used as crude extracts.	Minimum laccase selling price (MLSP) as low as USD 0.05/kU (highly competitive).	**(+)** Circular waste valorisation; media costs drop to 31–33% of total. **(−)** Variable batch quality; downstream recovery & mass-transfer limitations.	[142,143,144]
**Recombinant Heterologous Expression** (**TRL: 6–7**)	• Recombinant *Pichia pastoris* (glycerol feed). • High purity; Homogeneous single-cell suspension (bypassing mycelial-clumping viscosity, long fermentation cycles, and co-secretion of native isoenzyme mixtures)	EUR 0.34/kU (commercially viable for bulk).	**(+)** Exceptional volumetric productivity; allows protein engineering of robust mutants. **(−)** CAPEX for bioreactors; complex post-translational glycosylation control.	[40,43,145]
**Biomimetic Nanozymes** (**TRL: 3**)	• Self-assembled copper-organic frameworks (Cu-MOFs). • Crystalline, robust inorganic structure.	16.2-fold cheaper than equivalent mass of natural laccase.	**(+)** Outstanding thermal stability (>80% at 90 °C); 37.2-fold increase in catalytic rate. **(−)** Premarket nanosafety approval required; risk of metal-ion migration into food.	[100,101]

^‡^ TRLs are defined according to the EU Horizon 2020 framework: TRL 3, lab proof of concept; TRL 4, lab validation (simulated tests); TRL 5–6, validation/demonstration in industrially relevant environment; TRL 7, operational line prototype; TRL 8, safety-certified system; TRL 9, competitive manufacturing.

## Data Availability

No new data were created or analysed in this study. Data sharing is not applicable to this article.

## Abbreviations

The following abbreviations are used in this manuscript:

ABTS	2,2′-azino-bis(3-ethylbenzothiazoline-6-sulfonic acid)
BSA	Bovine Serum Albumin
CA	Caffeic acid
Cu-MOF	Copper-based Metal–Organic Framework
DPPH	2,2-diphenyl-1-picrylhydrazyl
EAB	Elongation At Break
FA	Ferulic acid
GA	Gallic acid
Gox	Glucose oxidase
GT	Gallotannins
GRAS	Generally Recognised As Safe
HP	4-hexyloxyphenol
HQ	Hydroquinone
MLSP	Minimum Laccase Selling Price
MW	molecular weight
NADES	Natural Deep Eutectic Solvent
NIAS	Non-Intentionally Added Substances
OPEX	Operational Expenditures
Pes	Pickering emulsions
SLAC	*Streptomyces coelicolor* laccase
TTC	Threshold of Toxicological Concern
TTI	Time-Temperature Indicator
TOS	Total Organic Solids
TRL	Technology Readiness Level
TS	Tensile Strength
